# Effects of Low Level Laser Therapy on Ovalbumin-Induced Mouse Model of Allergic Rhinitis

**DOI:** 10.1155/2013/753829

**Published:** 2013-11-11

**Authors:** Binhye Choi, Mun Seog Chang, Ha Young Kim, Jae-Woo Park, Bongha Ryu, Jinsung Kim

**Affiliations:** ^1^Department of Internal Medicine, College of Korean Medicine, Kyung Hee University, 26 Kyungheedae-ro, Dongdaemun-gu, Seoul 130-701, Republic of Korea; ^2^Department of Prescriptionology, College of Korean Medicine, Kyung Hee University, 26 Kyungheedae-ro, Dongdaemun-gu, Seoul 130-701, Republic of Korea

## Abstract

*Introduction*. This study was designed to investigate the effects of low level laser therapy (LLLT) on experimental allergic rhinitis (AR) models induced by ovalbumin. *Materials and Methods*. AR was induced by 1% ovalbumin in mice. Twenty-four mice were divided into 4 groups: normal, control, low, and high dose irradiation. Low and high dose LLLT were irradiated once a day for 7 days. Total IgE, cytokines concentrations (IL-4 and IFN-**γ**), and thymus and activation regulated chemokine (TARC) were measured. Histological changes in the nasal mucosal tissue by laser irradiation were examined. *Results*. LLLT significantly inhibited total IgE, IL-4, and TARC expression in ovalbumin-induced mice at low dose irradiation. The protein expression level of IL-4 in spleen was inhibited in low dose irradiation significantly. IL-4 expression in EL-4 cells was inhibited in a dose dependent manner. Histological damages of the epithelium in the nasal septum were improved by laser irradiation with marked improvement at low dose irradiation. *Conclusion*. These results suggest that LLLT might serve as a new therapeutic tool in the treatment of AR with more effectiveness at low dose irradiation. To determine the optimal dose of laser irradiation and action mechanisms of laser therapy, further studies will be needed.

## 1. Introduction

Allergic rhinitis (AR) is a condition characterized by one or more of the following nasal symptoms: congestion, rhinorrhea (anterior and posterior), sneezing, and itching [1], which is estimated to affect up to 40% of the population worldwide [2]. At present, some pharmacological therapies like antihistamines, corticosteroids, decongestants, and mast cell stabilizers are available for the AR patients [3]; however, AR symptoms are not always satisfactorily controlled by medication, and some patients fail to respond to conventional treatments [4].

Acupuncture is one of the most popular complementary and alternative medical treatments and classified according to stimulating materials or methods such as metal needle, laser stimulation, or herbal extracts (pharmacopuncture) [5]. In case of AR, acupuncture treatment has been reported to show beneficial effects through clinical trials. Moreover, EX-HN9 (*內迎香*) is used as one of the main acupuncture points for its treatment [6]. However, there are some controversies about the effectiveness of acupuncture treatment on AR and some limitations for applying acupuncture in the clinical field (e.g., pain or discomfort due to needle pain). Therefore, another modality of acupuncture to improve the effectiveness on AR and compliance with treatment is necessary to be investigated.

Low level laser therapy (LLLT) has been clinically used to aid wound healing [7], relieve pain in musculoskeletal diseases [8, 9], and stimulate acupuncture points [10], and its anti-inflammatory and immunosuppressive effects have been examined in the laboratory [11–13]. However, there are few studies that have examined the effects of LLLT on AR.

In the present study, to investigate the effects of laser acupuncture on AR, the ovalbumin (OVA) induced allergic rhinitis model was used. Particularly, we irradiated the low level laser into the intranasal cavity of the mouse for recreating the laser acupuncture on EX-HN9 according to the dose of irradiation. The serological parameters of allergic inflammation including IgE and cytokines (IL-4 and IFN-*γ*) and thymus and activation regulated chemokine (TARC) were examined with the *in vitro *detection of IL-4 expression in Th2 cell as well. Histological examination was also carried out to measure the changes on inflammatory cell infiltration of intranasal epithelium by LLLT.

## 2. Materials and Methods

### 2.1. Laser

The laser device made by Gwangju Institute of Science and Technology (Gwangju, Korea) had the following characteristics: low-power laser of 658 nm infrared wavelength, aluminum gallium indium phosphide (InGaAlP) semiconductor, fiber diameter of material (Core/Cladding) as 50/125 *μ*m, and 30 mW of output. Under anesthesia and with maximal exposure of mouse nostril to laser beam, the LLLT to mice was conducted for irradiating overall mucosal surface of nasal cavity including EX-HN9. The laser was irradiated 10 mm apart from each nostril of mouse with vertical setting of the laser device. The parameters of laser irradiation in the present study were shown in Table 1.

### 2.2. Th2 Cell Culture and Cell Viability

Murine thymic lymphoma cells (EL-4) were purchased from the KoreaCell Line Bank, Seoul, Korea. EL-4 cell lines were cultivated in RPMI-1640 medium with extra L-glutamine (4 mM), sodium pyruvate (1.0 mM), 4.5 g L^−1^ glucose, penicillin (100 U/mL), streptomycin (100 U/mL), and 10% v/v fetal bovine serum. For differentiation of EL-4 cells, phorbol-12-myristate-13-acetate (PMA) and 4-tert-Octylphenol (OP) (Sigma-Aldrich, MO, USA) were treated at a density of 5 × 10^5^ cells/well. Control cells were incubated without MPA or OP. Cells were collected by gently scraping (cell scraper) in culture medium and centrifuged for 8 min at 300 g, and the supernatant was removed. Cell pellets were washed twice in ice cold PBS and directly analyzed or stored at −80°C for further analysis for RT-PCR. Optimal PMA and OP concentration was selected by determining the percentage of differentiated cells and their viability. Laser irradiation was delivered to the culture plate from above via an optical fiber. The laser beam was clipped to cover the entire area of the plate using cap. The laser densities were 100 mJ (30 mW, 32 s), 500 mJ (30 mW, 160 s), 1,000 mJ (30 mW, 320 s), and 2,000 mJ (30 mW, 640 s).

### 2.3. Animal and Grouping

All experiments were carried out in accordance with the guidelines of Kyung Hee University for animal care. Male Balb/c mice, aged 6 weeks and weighing 18–20 g, were randomly divided into 4 groups: normal (OVA untreated and no irradiation, *n* = 6), control (treated only OVA, *n* = 6), low dose irradiation (1,000 mJ irradiation, *n* = 6), and high dose irradiation (2,000 mJ irradiation, *n*  =  6).

### 2.4. ELISA Assay

Serum of OVA mouse was separated from the whole blood collected by the cardiac puncture and stored in the deep-freezer for the serum IgE assay. After the final laser irradiation, spleen tissues were kept in coldlysis buffer (Cell signaling, Carlsbad, CA, USA) for IL-4, INF-*γ*, and TARCELISA assay. IgE, IL-4, INF-*γ*, and TARCELISA kits (R&D Systems, Inc., Minneapolis, MN, USA) were used and the cytokine concentrations were assessed according to the manufacturer's cytokine ELISA protocol.

### 2.5. OVA Sensitization and Challenging

**Figure 1:**
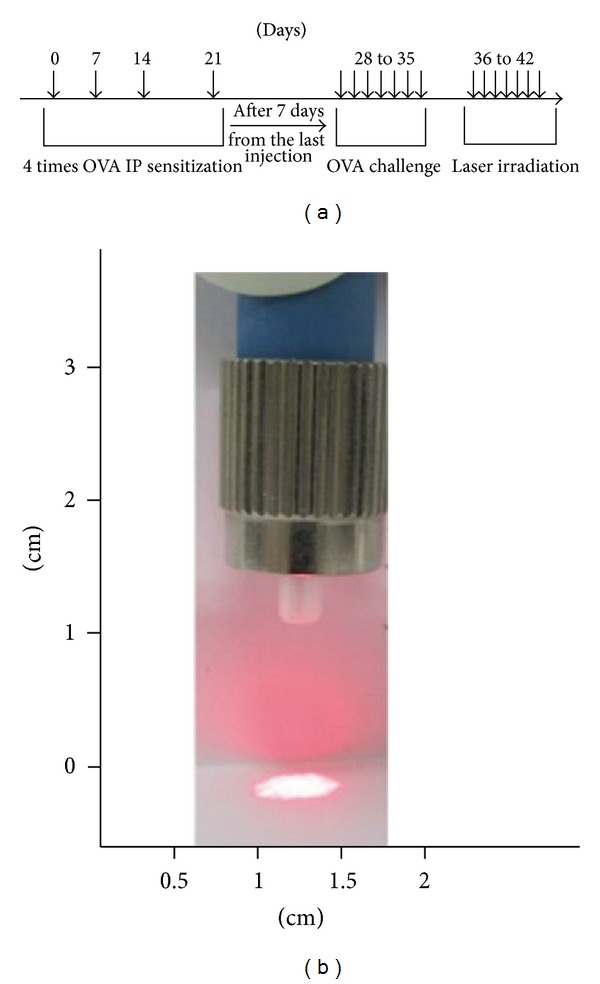
(a) Protocol for sensitization of ovalbumin (OVA). Sensitization was performed by intraperitoneal injection of OVA on days 0, 7, 14, and 21. After 1 week later, mice were challenged OVA aerosol daily for 1 week to induce local sensitization. Next day, laser was irradiated daily for 1 week. Normal group mice were injected and challenged with PBS instead of OVA. (b) Laser instrument used for the intranasal irradiation.

Through allergen exposure using the chronic exposure protocol, mice were sensitized and challenged, shown in Figure 1. Briefly, 6 week-old mice were systemically sensitized to OVA (10 *μ*g/0.1 mL chicken egg albumin, ovalbumin, grade V, 98% pure, Sigma, St. Louis, MO, USA) by intraperitoneal (i.p.) injection on days 7, 14, and 21. Exposures to aerosolized OVA (10 mL of 10 mg OVA/mL 1% saline solution) were commenced on day 28 after the first i.p. (Figure 1). In this AR model, 1,000 mJ (30 mW, 320 s) and 2,000 mJ (30 mW, 640 s) laser were irradiated once a day for 7 days.

### 2.6. Western Blot

All spleen samples were lysed in ice-cold lysis buffer containing Tris-HCl (pH 7.4) (20 mM), 1% Triton X-100, 0.1% SDS, EDTA (2 mM), and phenylmethyl sulfonyl fluoride (PMSF) (1 mM). The amount of protein in each sample was determined using the Bradford assay. The samples were subjected to 10% sodium dodecyl sulfate polyacrylamide gel electrophoresis (SDS-PAGE). The protein spots were electrotransferred to a polyvinylidenedifluoride (PVDF) membrane. The membrane was incubated with block buffer (PBS containing 0.05% Tween-20 and 5% w/v nonfat dry milk) for 1 h, washed with PBS containing 0.05% Tween-20 (PBST) three times, and then probed with IL-4 antibody (1 : 1,000 diluted, Cell Signaling Technology, USA) overnight at 4°C. In addition, the intensity of the blots probed with 1 : 1,000 diluted solution of antibody *β*-actin (Cell Signaling Technology, USA) was used as the control to ensure that a constant amount of protein was loaded into each lane of the gel. The membrane was washed for 5 min (3 times) in PBST, shaken in a solution of HRP-linked anti-rabbit IgG secondary antibody (1 : 2,000 diluted) for 2 h at RT, and again washed for 5 min (3 times) in PBST. The expressions of proteins were detected by enhanced chemiluminescent (ECL) reagent (Millipore, Billerica, MA, USA).

### 2.7. RT-PCR

First strand cDNA synthesis with 5 *μ*g of total RNA was performed using MMLV reverse transcriptase and oligodT primer for 1 h at 42°C. Subsequently, the PCR-amplification was performed by a modified method originally described by Saiki et al. [14]. Firstly, 5 *μ*L of cDNA was added to 2.5 *μ*L of 10x PCR buffer, 1 *μ*L of 25 mM MgCl_2_, 1 *μ*L of 2.5 mM dNTP, 0.5 *μ*L of polymerase (1 U), 1 *μ*L of each primer (4 pmol), and DEPC-H_2_O to give a final volume of 25 *μ*L. The sequences of IL-4 primers were as follows: 5′-TAGTTGTCATCCTGCTCTT-3′ as forward primer and 5′-CTACGAGTAATCTTGC-3′ as reverse primer. PCR-products were separated on a 1.5% agarose gel, visualized by ethidium bromide using i-MAX gel image analysis system (CoreBioSystem, Seoul, Korea), and analyzed using Alpha Easy FC software (AlphaInnotech Corporation, San Leandro, CA, USA). Results from at least three separate experiments were used for statistical analysis.

### 2.8. Hematoxylin and Eosin (HE) Staining

For histological studies, paraffin-embedded tissue sections (5 *μ*m thick, coronal section) were stained by HE staining. The sections were deparaffinized and rehydrated in xylene, 100, 95, 80, and 70% ethanol. The sections were overstained with hematoxylin, usually 3–5 min, and rinsed off excess stain in deionized water. Then they were destained a few seconds in acidic alcohol until sections look red, usually 4-5 dips, and rinsed briefly in deionized water to remove the acid. Hematoxylin stained slides from the last tap water were rinsed and placed in 70% ethanol for 3 min. Slides were placed in eosin for 2 min and taken slides through 95 and 100% ethanol and xylene. After HE staining, slides were mounted with Canada balsam.

### 2.9. Statistical Analysis

All quantitative data derived from this study were analyzed statistically. The results were expressed as the mean ± SEM. Differences between groups were assessed by one-way ANOVA using the SPSS version 12.0 software package for Windows (SPSS Inc., Chicago, IL, USA). Statistical significance at *P* < 0.05 has been given respective symbols in figures.

## 3. Results

### 3.1. EL-4 Cell Differentiation Condition and Cell Viability

Before investigating the effects of LLLT on the AR mice, cell viability was assessed upon various doses of irradiation (100, 500, 1,000, and 2,000 mJ) to determine the irradiating doses of laser. As a result, laser treatments up to 1,000 mJ did not induce any changes in terms of cell cytotoxicity. After 2,000 mJ of laser irradiation, cell viability was decreased than that of untreated cell; however, there was no significance (data not shown). Based on this, doses for animal experiments were chosen as 1,000 mJ as low dose irradiation and 2,000 mJ as high dose irradiation, respectively. According to the scheme as shown in Figure 1, 3 groups of animals were exposed and treated as indicated.

### 3.2. Effects of LLLT on Total IgE Level in the OVA-Induced AR Mice Serum

**Figure 2:**
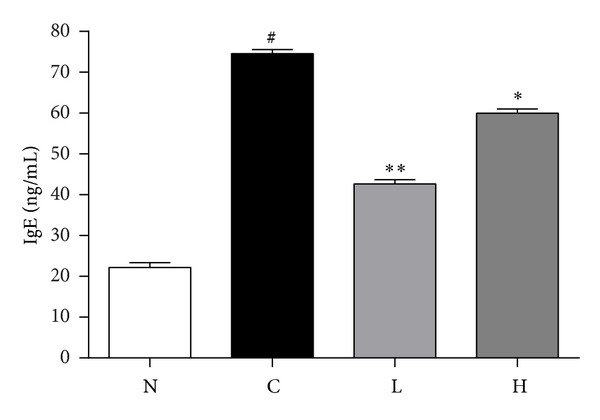
Measurement of serum total IgE level. Level of total IgE was expressed as optical density (OD) at 450 nm. N: normal group (no stimulation), C: control group (stimulation by ovalbumin without irradiation), L: low dose irradiation on control group (30 mW/320 s), and H: high dose irradiation on control group (30 mW/640 s). ^#^ *P* < 0.05 versus the normal group. *P < 0.05 versus the control group. **P < 0.01 versus the control group.

To further analyze the allergic response, we measured the serum levels of total IgE. The concentration of total IgE level was significantly increased in control group (74.54 ± 1.05%) compared to that of normal group (22.17 ± 1.21%, Figure 2). The total IgE levels of low dose irradiation group (42.64 ± 1.05%) and high dose irradiation group (59.93 ± 1.10%) were significantly decreased than those control group, respectively (Figure 2).

### 3.3. LLLT Reduces Cytokines IL-4 and TARC Production

**Figure 3:**
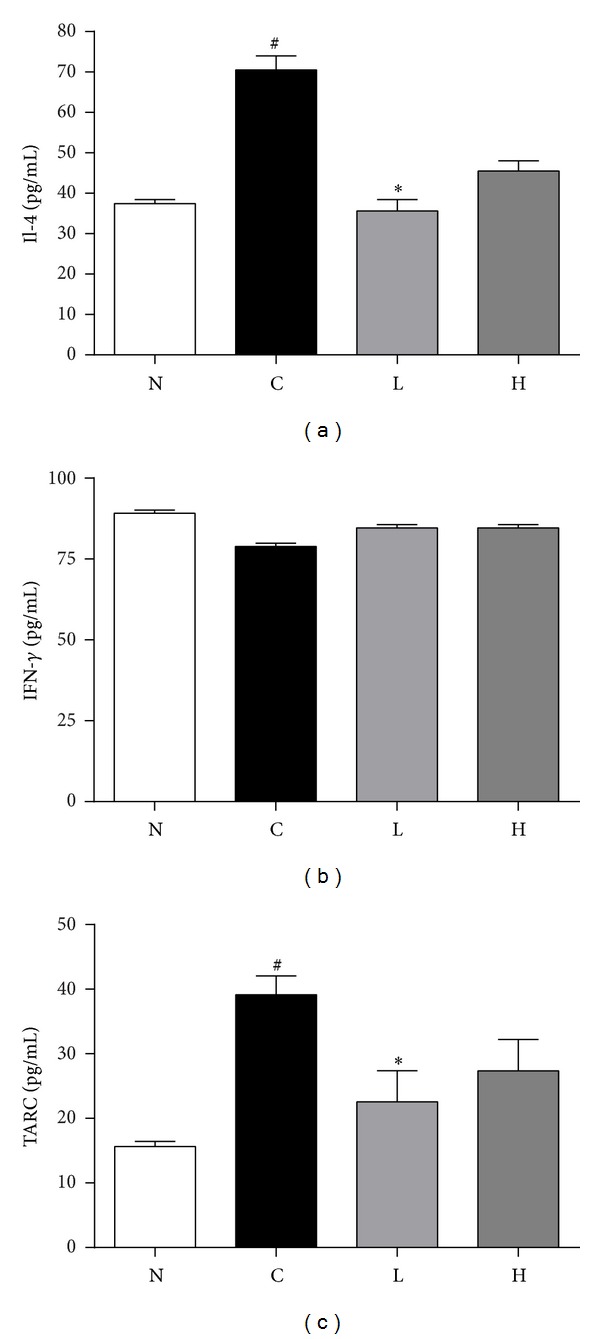
Measurement of serum cytokines production. Serum cytokine levels of IL-4 (a), TARC (b), and IFN-*γ* (c) were presented. Levels of IL-4, TARC, and IFN-*γ* were expressed as optical density (OD) at 450 nm. N: normal group (no stimulation), C: control group (stimulation by ovalbumin without irradiation), L: low dose irradiation on control group (30 mW/320 s), and H: high dose irradiation on control group (30 mW/640 s). ^#^ *P* < 0.05 versus the normal group. *P < 0.05 versus the control group.

To further examine the effect of LLLT on AR, the production of cytokines was evaluated through the ELISA test. Serum concentrations of IL-4, TARC, and IFN-*γ* were determined and are shown in Figure 3. The IL-4 level in control group (70.48 ± 3.49%) showed elevated production of IL-4 compared to that in normal group (37.40 ± 1.01%), while the low dose irradiation group (35.65 ± 2.77%) and high dose irradiation group (45.50 ± 2.52%) were significantly decreased than that of control group (Figure 3(a)). In addition, TARC production in serum showed similar results as observed in IL-4 production. TARC level in control group (39.16 ± 2.90%) was increased than that in normal group (15.63 ± 0.78%), while the low dose irradiation group (22.56 ± 4.80%) and high dose irradiation group (27.36 ± 4.87%) were significantly decreased than that of control group (Figure 3(c)). The levels of IFN-*γ* production in normal, control, low dose irradiation, and high dose irradiation groups were 89.12 ± 1.00%, 78.91 ± 1.00%, 84.64 ± 1.01%, and 84.61 ± 1.01%, respectively. There was a slight difference between groups; however, there was no statistical significance (Figure 3(b)).

### 3.4. LLLT Reduces IL-4 Expression Levels in Mice Spleen and EL-4 Cells

**Figure 4:**
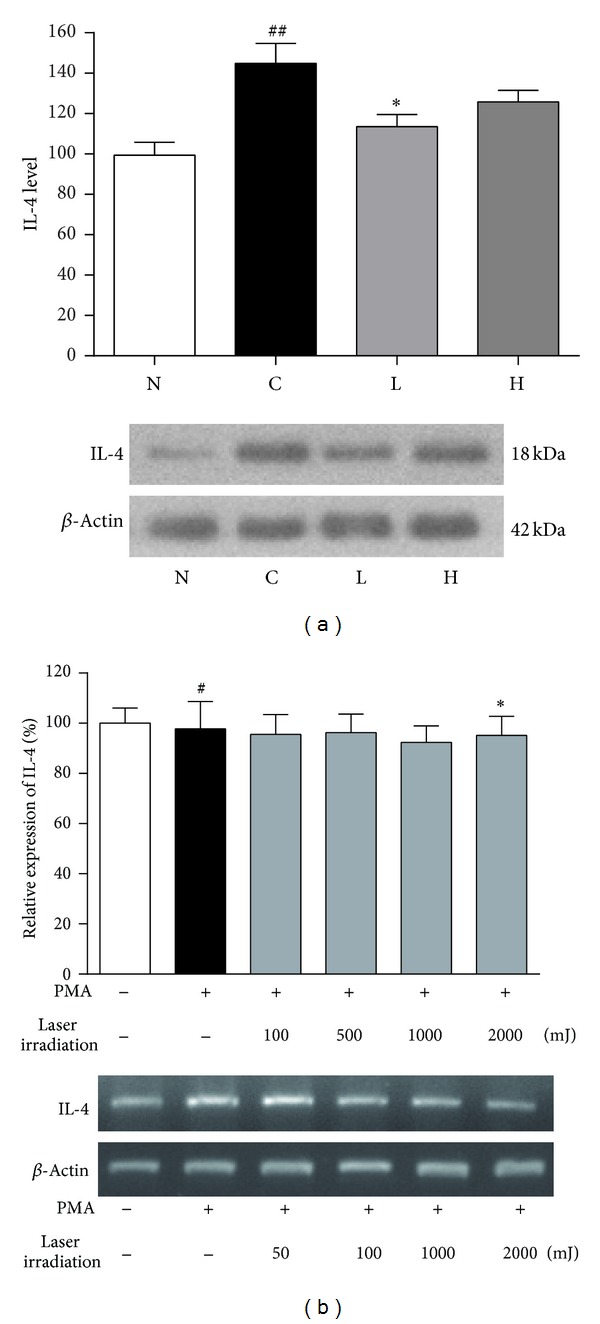
The IL-4 expression in mice spleen and EL-4 cells. (a) Protein expression levels of IL-4 in mouse spleen. The representative bands of IL-4 from electrophoresed gel were shown. N: normal group (no stimulation), C: control group (stimulation by ovalbumin without irradiation), L: low dose irradiation on control group (30 mW/320 s), and H: high dose irradiation on control group (30 mW/640 s). ^#^ *P* < 0.05 versus the normal group. ^##^ *P* < 0.01 versus the normal group. *P < 0.05 versus the control group. (b) mRNA levels of IL-4 in EL-4 cells. The representative bands of IL-4 from electrophoresed gel after RT-PCR reaction. Normal: no stimulation; control: stimulation by PMA. ^#^ *P* < 0.05 versus the normal group. *P < 0.05 versus the control group.

Western blot was performed to determine the effect of laser irradiation on protein expression in spleen of AR mice. *β*-Tubulin was used as internal control. As the result indicated in Figure 4(a), IL-4 protein level in control group (144.85 ± 9.82%) was increased as compared to normal group (99.41 ± 6.45%), while the IL-4 protein levels of low dose irradiation group (113.59 ± 5.95%) and high dose irradiation group (125.77 ± 5.74%) were significantly decreased than those of control group (Figure 4(a)). To confirm the effects of LLLT on IL-4 expression, we investigated the IL-4 mRNA level in the EL-4 cells. IL-4 expression of control group (112.43 ± 4.11%) was increased upon PMA treatment as compared to that of normal group (100.00 ± 5.43  %), while the laser irradiation groups (100, 500, 1,000 and 2,000 mJ) were decreased than those of control group (106.61 ± 8.46%, 92.98 ± 7.98%, 91.87 ± 8.97%, and 78.37 ± 9.53%, resp., Figure 4(b)).

### 3.5. Histological Changes after Laser Irradiation

**Figure 5:**
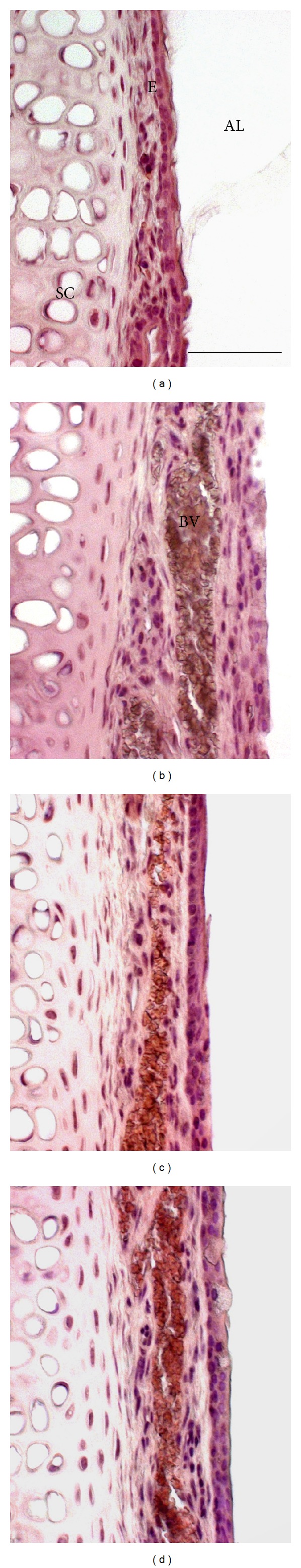
Hematoxylin and eosin staining of nasal septum. Mice were sensitized and challenged intranasally with no treatment (a), OVA treatment (b), 1,000 mJ laser-irradiation (c), and 2,000 mJ laser-irradiation (d). Images were obtained at an objective magnification of ×400. Scale bars = 100 *μ*m. Airway lumen (AL), epithelium (E), blood vessels (BV), and septum cartilage (SC) were shown, respectively.

HE staining was carried out to observe the histological changes of nasal septum. The respective numbers of inflammatory cells in the epithelium of nasal septum in the control group (Figure 5(b)) were significantly higher than those observed in the normal group (Figure 5(a)). Low dose irradiation group (Figure 5(c)) and high dose irradiation group (Figure 5(d)) showed reduced number of infiltrated inflammatory cells in the epithelium as well as recovered epithelium structure from OVA-induced epithelium damage. Especially, in low dose irradiation group, the effect of laser irradiation was most effective as compared to that observed in normal group (Figures 5(a) and 5(c)).

## 4. Discussion

Medical application of laser therapy has been classified into two categories: high level laser and low level laser. High level laser has a range between 3,000 and 10,000 mW and has been used in surgical purpose, while low level laser which has been applied as a laser acupuncture in traditional Korean medicine has below 1,000 mW of output [15]. Laser acupuncture is a painless, noninvasive treatment modality and presents no risk of infection. According to a literature review, evidence was found to support the use of laser acupuncture in the treatment of myofascial pain, postoperative nausea, and vomiting and for the relief of chronic tension headache [10]. It has been reported that low level laser stimulates certain types of cells and tissues using photoenergy, thus increases blood circulation, collagen synthesis, cell growth and bone regeneration and relieves inflammation, pain, and edema [16–22].

In this study, to investigate the effect of LLLT on animal model of AR, we determined the dose of laser irradiation at first, because targeting the acupuncture point of the nasal cavity as therapeutic purpose of laser treatment has not been tried yet. For this, EL-4 cells were subjected to viability assays by exposing to increasing doses of laser irradiation up to 2,000 mJ. Results showed that laser irradiation did not induce any notable cell death of EL-4 cell. Because the EL-4 is a cancerous cell, we could not exclude the possibility that the viabilities of these cells may be affected by higher rate of metabolic circumstances in cytoplasm. However, there were no morphological changes of EL-4 cells as observed in necrosis and cytotoxic environment. As a result, we applied two different doses of laser irradiation (1,000 and 2,000 mJ) to mouse AR model and examined serum level of IgE, cytokines (IL-4 and INF-*γ*), and TARC. In the low dose irradiation group, a significant inhibition of IgE, IL-4, and TARC was indicated. In addition, the expression of IL-4 was significantly inhibited in the low dose irradiation group, and the dose-dependent inhibition of EL-4 cells was detected. Particularly, inflammatory cell infiltration of nasal septum was decreased by laser irradiation in terms of histological examination with marked decrease in low dose irradiation group.

Type I hypersensitivity of AR is mediated by IgE immune response against foreign allergen and leads to the development of acute inflammation through IgE sensitive mast cells and other immune modulators [23]. When nasal mucosa is exposed to allergen, many immune cells such as mast cells, neutrophils, eosinophils, and lymphocytes are activated and cause infiltration of cells followed by blood vessel damage and histological change of nasal tissues [23].

IgE triggers allergic symptoms by activating the secretion of granules from mast cell and basophil through binding to its surface receptors [24]. Increasing IgE level in serum can induce acute hypersensitivity and subsequently cause symptoms for hay fever, asthma, and anaphylaxis. In our experiments, OVA-induced mouse showed increased IgE production in serum, and this was decreased by the low level laser irradiation, suggesting that LLLT has inhibitory effects on IgE production in the AR mouse model.

It has been known that IL-4 plays an important role in development of early stage of inflammation at AR, and it sustains the inflammatory process through the induction of Th2 cell differentiation and function [25, 26]. On the other hand, IFN-*γ* which is secreted from Th1 cells stimulates antibody production of B cells and induces IgG mediated immune responses [27]. It has been also reported that increasing level of IFN-*γ* could reduce the early and the late stage of allergic reaction and relieve the symptom [28, 29]. Thus, it has an antagonic action against IL-4 function and IgE production. TARC (CCL17) is a receptor expressed in Th2 cells and binds to CCL4 with high affinity [30]. It has been known to be involved in leukocytes migration and regarded as one of key inflammatory factors with IgE in atopic immune responses [24]. To investigate the effect of LLLT on cytokines production in OVA-induced AR mice, we performed ELISA assays. Results showed that the low level laser irradiation was significantly reducing serum IL-4 and TARC levels, while INF-*γ* level was not affected by any doses of LLLT. In the present study, the inhibitory effect of LLLT on IL-4 production was confirmed by RT-PCR analysis and western blot using mouse spleen and EL-4 Th2 cells, respectively. These results strongly suggest that the effect of LLLT on AR mice was derived from Th2 mediated immune responses by regulating IgE and IL-4 production rather than those of Th1 related immune responses. In addition, IL-4 is involved in synthesis and secretion of IgE by B cells. Thus, inhibiting the expression of IL-4 might be related to the serum levels of IgE. However, molecular mechanisms by which LLLT reduces the IgE and IL-4 production remain to be studied.

Histological change of nasal mucous membrane is an important indicative factor in AR development. Therefore, we examined whether the effect of LLLT is accompanied by histological improvement of epithelium of nasal septum. OVA-induced AR mice showed clear damages in nasal epithelium as observed by infiltration of cells across the blood vessel, and this was relieved by LLLT not only in number of infiltrated cells but also in the overall histological damages of nasal septum. Together with the effect of LLLT on the regulation of cytokines expression, anti-inflammatory effects of LLLT have been reported. In animal model of acute lung inflammation induced by intestinal ischemia/reperfusion or LPS, LLLT has relieved airway inflammation through the induction of IL-10 and reduction of TNF and macrophage inflammatory protein-2 (MIP-2) expression [31, 32]. Considering the maximal effects at low dose irradiation in the present study, the effective irradiation dose of LLLT should be determined when it subject to the application for the therapeutic use in human.

## 5. Conclusion

LLLT might be an alternative therapeutic method for treating AR or its symptoms, and low dose irradiation (1,000 mJ) could be more effective than high dose irradiation (2,000 mJ). This could be explained by the function of low level laser irradiation that inhibits the progression of AR through the regulation of Th2 cell activation, differentiation, and expression of inflammatory molecules such as cytokines and IgE. To explain the clinical effectiveness, optimal doses of laser irradiation, or the detailed mechanisms, further investigations including clinical applications to human or molecular studies will be needed.

## Figures and Tables

**Table 1:** Laser irradiation parameters involved in this study.

Parameters	Values
Power output	30 mW
Wavelength	658 nm
Mode of action	Continuous
Fiber diameter (Core/Cladding)	50/125 um

Cell	
Power density	3.157 × 10^−3^ W/cm^2^
Spot size	9.5 cm^2^
Irradiation time	32 s (100 mJ/cm^2^), 160 s (500 mJ/cm^2^), 320 s (1000 mJ/cm^2^), 640 s (2000 mJ/cm^2^)

Mouse	
Power Density	15 × 10^−2^ W/cm^2^
Spot size	0.2 cm^2^
Irradiation time	320 s (1000 mJ/cm^2^), 640 s (2000 mJ/cm^2^)
